# Can CT Screening Give Rise to a Beneficial Stage Shift in Lung Cancer Patients? Systematic Review and Meta-Analysis

**DOI:** 10.1371/journal.pone.0164416

**Published:** 2016-10-13

**Authors:** Zixing Wang, Yaoda Hu, Yuyan Wang, Wei Han, Lei Wang, Fang Xue, Xin Sui, Wei Song, Ruihong Shi, Jingmei Jiang

**Affiliations:** 1 Institute of Basic Medical Sciences, Chinese Academy of Medical Sciences/School of Basic Medicine, Peking Union Medical College, Beijing, China; 2 Peking Union Medical College Hospital, Chinese Academy of Medical Science, Beijing, China; 3 National Institutes for Food and Drug Control, State Food and Drug Administration, Beijing, China; Dartmouth College Geisel School of Medicine, UNITED STATES

## Abstract

**Objectives:**

To portray the stage characteristics of lung cancers detected in CT screenings, and explore whether there’s universal stage superiority over other methods for various pathological types using available data worldwide in a meta-analysis approach.

**Materials and Methods:**

EMBASE and MEDLINE were searched for studies on lung cancer CT screening in natural populations through July 2015 without language or other filters. Twenty-four studies (8 trials and 16 cohorts) involving 1875 CT-detected lung cancer patients were enrolled and assessed by QUADAS-2. Pathology-confirmed stage information was carefully extracted by two reviewers. Stage I or limited stage proportions were pooled by random effect model with Freeman-Tukey double arcsine transformation.

**Results:**

Pooled stage I cancer proportion in CT screenings was 73.2% (95% confidence interval: 68.6%, 77.5%), with a significant rising trend (*P*_trend_<0.05) from baseline (64.7%) to ≥5 repeat rounds (87.1%). Relative to chest radiograph and usual care, the increased stage I proportions in CT were 12.2% (*P*>0.05), and 46.5% (*P*<0.05), respectively. Pathology-specifically, adenocarcinomas (66%) and squamous cell lung cancers (17%) composed the majority of CT-detected lung cancers, and had significantly higher stage I proportions relative to chest radiograph (bronchioloalveolar adenocarcinomas, 80.9% vs 51.4%; other adenocarcinomas, 58.8% vs 38.3%; squamous cell lung cancers, 52.3% vs 38.3%; all *P*<0.05). However, the percentage of small cell lung cancer was lower using CT than other detection routes, and no significant difference in limited stage proportion was observed (6.8% vs 10.8%, *P*>0.05).

**Conclusion:**

CT screening can detect more early stage non-small cell lung cancers, but not all of them could be beneficial as there are a considerable number of indolent ones such as bronchioloalveolar adenocarcinomas. Still, current evidence is lacking regarding small cell lung cancers.

## Introduction

With over one million annual fatal cases [1], lung cancer is incontestably the leading cancer burden worldwide. Historically, there has been long-standing interest in the early detection of pulmonary malignancy for the improvement of treatment effectiveness and prognosis [2]. Consequently, an extensive number of studies focusing on the efficacy and feasibility of mass screening have been or are being conducted [3–26]. Among these efforts, the National Lung Screening Trial (NLST) is the first and only randomized controlled trial (RCT) to date demonstrating that lung cancer mortality can be reduced by conducting computed tomography (CT) mass screening [15]. Radical screening practices have been explored, accompanied by criticisms concerning high false positive rates, screening related anxiety, and cost-effectiveness [27], and questions remain about technical issues such as selection criteria and effective nodule management protocols [28]. However, before balancing all of these benefits and potential adverse effects and before carrying out solutions to barriers in implementing a screening program [28], one fundamental key issue, namely the stage characteristics and stage superiority over other approaches of CT screen-detected cancers, has been raised but still not fully explored [20,29], which is the theoretical precondition of any screening benefits [30].

Previous studies showed a less intention or weak capability to depict a comprehensive view of the cancer stage distribution as the cancer case numbers are generally small [3–26]. The purpose of this systematic review is to shed light on the stage characteristics of subjects screened for lung cancers with CT, by using meta-analysis approach to synthetize available data from existing screening programs. The analysis process is organized by the following order: First, we attempted to address two basic questions: (a) How frequently are early stage cancers detected using CT, with regard to different populations and screening schemes? (b) Does the baseline and repeat screening rounds show different stage distribution patterns, and is there any changing trend over time as the screening program proceeds? After this descriptive analysis process, we go on to evaluate: (c) To what extent can CT detect more early stage cancers over other cancer detection routes? And then by pathology-specific analysis explore the most topical and important question of: (d) Do the additionally detected early lung cancers represent a real stage shift, or simply a mirage of over-diagnosis[31] (which denotes that, according to Patz et al [32], more than 18% lung cancers detected by CT in the NLST were excessively diagnosed indolent cancers compared with chest radiograph, and this rate was even much higher, namely 30%-65% according to Young et al [33], in European trials when compared with usual care).

## Materials and Methods

The report of this study follows the Preferred Reporting Items for Systematic Reviews and Meta-analyses (PRISMA) statement guidelines (PRISMA checklist in S1 Table).

### Information Source and Search Strategy

Cohort (uncontrolled) or randomized controlled designed studies that reported the use of CT for lung cancer screening, in natural populations of any age, without language restriction, were considered in this systematic review and meta-analysis. To identify potential studies, an expert librarian was consulted in developing search strategy, and two reviewers (ZW and YH) conducted a comprehensive electronic search in MEDLINE and EMBASE databases from January 1990 (the year CT was introduced for lung cancer screening) through July 2015. The index words “lung cancer”, “CT”, “screening”, and their synonyms (obtained by referring to their Mesh terms) with the field names “title” or “abstract”, were used without language or other filters (a full record of the electronic search is available in S1 Text). References regarding related articles from the literature already identified were further reviewed. Authors and their affiliations, and the names of the identified screening programs were checked to obtain up to date results.

### Eligibility Criteria and Study Selections

Lung cancer screening studies using CT were considered which: (a) were conducted in a community based, hospital centered, or nationwide population; (b) reported at least ten lung cancers; and (c) provided pathologically confirmed stage information (by bronchoscopic, aspiration, surgical biopsies, etc.) for the baseline screen, repeat rounds or as a whole (as clinical staging overestimates early cancer proportions and will introduce heterogeneity in pooled estimates). Studies were excluded if they: (a) focused on special occupational groups (exposed to asbestos, mineral dust, or nuclear fuel), or among patients such as those with HIV infections or tuberculosis diseases; (b) took CT as a subsequent examination (e.g. CT screening in chest radiograph negative subjects); or (c) failed to obtain pathological diagnoses for the screen detected lung cancers after contacting authors twice (actually only one author out of five studies responded but could not provide this information). Two reviewers (ZW and YH) did the study selections and reached a perfect agreement on study enrollment result when comparing the studies against the explicit eligibility criteria listed above.

### Data Extraction and Quality Assessment

With a piloted and refined data collection form on five randomly-selected studies, basic study information (author, initial screening year, design), population characteristics (actual number screened, age range, male proportion, smoking status criteria), screening protocol (number of maximum round, threshold for further examination and biopsy, and other criteria for suspicious nodules), individual patient data (gender, age and nodule size at the time of detection), and the outcome of interest (stage and pathology-specific stage information concerning both lung cancer patients and nodules, in both baseline and repeat rounds) were carefully extracted by two reviewers (ZW and YH, shown in S2 Table), first individually and then by discussion to resolve discrepancies. Study quality assessment was carried out using a QUADAS2 instrument (which measures the risks of bias and concerns of applicability in the domains of Patient Selection, Index Test, Reference Standard, and Flow and Timing) [34], independent of the data extraction process, and also in the form of double reviewers (by ZW and YH); a third input from LW was used when unsolved discrepancies occurred. Specific index questions are available in S3 Table.

### Summary Measures and Statistical Analysis

As there have been no significant changes in the definition of stage I lung cancer in the TNM staging system from version five to seven [35], we used pathological stage I (IA and IB were summarized) proportion as the main summary measure for representing early stage cancers (stage II-IV were not analyzed due to variations in the different staging systems applied by included studies). Small cell lung cancers were excluded from this calculation unless otherwise specified, but were all included and classified as ‘limited’ or ‘expansive’ stage for pathology-specific analysis. For patients with two or more cancers, patient level data were used to assess the screening outcomes in the public health perspective, and nodule level data was used for the pathological analysis. Specially, we maintained in this report the classification of bronchioloalveolar adenocarcinoma (BAC) as a special subgroup of adenocarcinomas though this concept was discontinued since 2011 in the IASLS/ATS/ERS Classification of lung adenocarcinoma [36], because almost all (but one) studies included in this analysis was initialed before that change and it was not practical to reclassify the BACs.

Fisher’s exact probability method was applied to obtain the 95% confidence interval (95% CI) for proportions. The Freeman-Tukey double arcsine transformation was applied in consideration of the fact that some proportions were close to the margins [37]. As heterogeneity in diagnosis/screening studies were perceived to be high (also tested using *I*^2^ index), random effect model was used to obtain conservative pooled estimates, and subgroup analysis of heterogeneity sources concerning study level information (region, design, quality, population age and smoking criteria), and patient level information (gender, age, and nodule size) were conducted. Multi-level logistic regression was used for the patient level data analysis. Additionally, Egger’s test was performed to explore any small-study effect/publication bias. *P* values smaller than 0.05 were considered significant. All statistical analyses were planned by a senior statistician (JJ) and performed by YW and WH using STATA 13.0 software.

## Results

### Inclusion of studies and basic results

**Fig 1:**
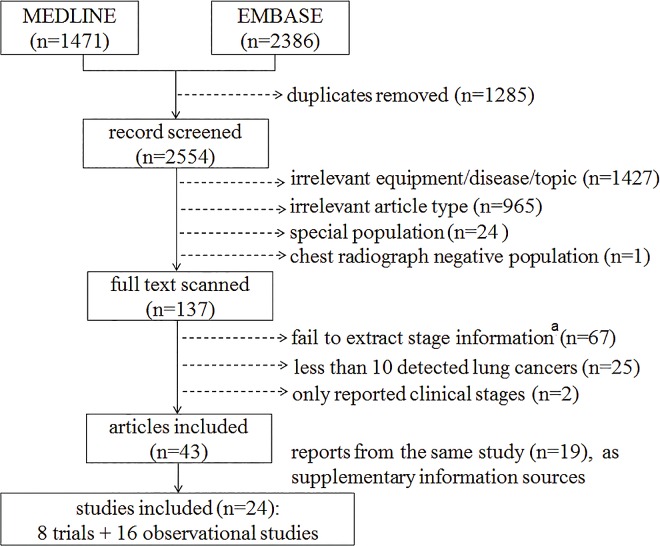
Study selection flow chart. A list of the full-text excluded articles are available in S2 Text. ^a^ We excluded the two summary reports from the International Early Lung Cancer Action Program and another two reports of its individual sites because only clinical stage information could be obtained, which is heterogeneous to the pathological staging method and could overestimate the estimates in this study.

The search strategy initially yielded 3839 articles. Exclusion and selection processes are detailed in Fig 1. After de-duplication (in 1285) and exclusion (in 2511), a total of 24 CT screening studies from 43 reports were enrolled (reporting on different screening rounds), including 16 cohort studies and eight RCTs that compared CT with chest radiograph (in 2) or usual care (in 6). These 24 programs (involving 105,007 CT screening participants and 1875 detected lung cancer patients) covered a wide geographic distribution, namely Asia (Japan, Israel, South Korea, and China), Europe (Germany, Spain, Italy, Netherlands, Belgium, Denmark, and Poland), and North America (United States and Canada). The basic characteristics of the included studies are outlined in Table 1. Study qualities were generally acceptable (ten ranked as high, 12 as moderate and only two as low quality for the study purpose; specific rankings are in S4 Table).

**Table 1:** Basic characteristics of the included CT lung cancer screening studies.

Study	Initial year	Study type	N of baseline CT	Male proportion (%)	Age(year)range	Smoking criteria	Control	Maximum round	Quality
ALCAP[3]	1993	cohort	2120	87	40+	20PY+/hemoptysis within 6m	-	22	moderate
Münster[4]	1995	cohort	817	72	40–78	20PY+	-	6	moderate
Mobile[5]	1996	cohort	7847	55	NA	ND	-	3	moderate
Israel[6]	1998	cohort	842	57	40+	smoker or former smoker	-	5	moderate
Hitachi[7]	1998	cohort	7956	79	50–69	ND	-	2	high
Mayo[8]	1999	cohort	1520	52	50+	20PY+,C<10y	-	5	high
Samsung[9]	1999	cohort	6406	86	45+	ND	-	4	high
Spain[10]	2000	cohort	911	74	40+	10PY+	-	>1	low
Milan[11]	2000	cohort	1035	71	50–84	20PY+,C<10y	-	10	moderate
LSS[12]	2000	RCT	1660	59	55–74	30PY+,C<10y	CR	2	high
DANTE[13]	2001	RCT	1276	100	60–74	20PY+,C<10y	usual care	5	high
PLuSS[14]	2002	cohort	3642	51	50–79	0.5p/d*25year,C<10y	-	2	low
NLST[15]	2002	RCT	26,722	59	55–74	30PY+,C<15y	CR	3	high
Toronto[16]	2003	cohort	3352	46	50–80	10PY+	-	6	moderate
Zhuhai[17]	2003	cohort	3582	74	40+	ND	-	7	moderate
NELSON[18]	2003	RCT	7155	84	50–75	15/d*25y/10/d*30y,c<10y	usual care	3	high
ITALUNG[19]	2004	RCT	1406	65	55–69	20PY+	usual care	4	moderate
DLCST[20]	2004	RCT	2052	56	50–70	20PY+,C<10y	usual care	5	moderate
COSMOS[21]	2004	cohort	5202	66	50+	20PY+,C<10y	-	2	moderate
MILD[22]	2005	RCT	2376	68	49+	20PY+,C<10y	usual care	6	moderate
LUSI[23]	2007	RCT	2029	50	50–69	15/d*25y/10/d*30y	usual care	5	high
CAMS-h[24]	2007	cohort	4690	70	40–88	ND	-	1	high
PLCSP[25]	2009	cohort	8649	52	50–75	20PY+	-	>1	high
Massachusetts[26]	2012	cohort	1760	52	50–74	20PY+	-	1	moderate

Abbreviations: RCT: randomized controlled trials; PY: pack year; C: smoking cessation; ND: not defined; CR: chest radiograph. The number of small cell cancers included when calculating the proportion of stage I cancer using CT: ALCAP, 4; Milan, 7; COSMOS, 6; LUSI, 3.

The summary estimate for the proportion of pathological stage I cancers was 73.2% (95% CI: 68.6%, 77.5%), with a significant increase from the baseline screens to the repeat rounds (70.1% and 75.6%, respectively; *P*<0.05). Analysis with the Egger’s test did not reveal any small-study bias in baseline (*P* = 0.698), repeat rounds (*P* = 0.711), or summary result (*P* = 0.613). However, because studies were characterized with various traits, all of these estimates suffer from great heterogeneity (all *I*^2^>50%).

Further subgroup analyses identified some potential heterogeneity sources (Tables 2 and 3). In the study level analysis, lower proportions of stage I cancer were reported in studies that adopted a RCT design (compared with uncontrolled cohorts, 66.8% vs 77.0%), or in studies that were conducted in North America (63.0%, compared with in Europe: 73.0%, or compared with in Asia: 83.5%), or in studies whose study populations were restricted to those aged ≥50 years (compared with no age limits, 69.8% vs 80.2%), or studies that were only conducted in smokers/ex-smokers (compared with no smoking status limits, 70.5% vs 83.5%), all *P*<0.05. No heterogeneity was observed between studies that were initiated before and after the year of 2000, and no heterogeneity was seen among studies that were rated as high, medium or low qualities. Generally, separate subgroup analyses on baseline and repeat rounds showed similar results, though the statistical inferences were instable due to smaller sample sizes.

**Table 2:** Study level subgroup analysis of sources of heterogeneity regarding the proportion of stage I cancers in CT lung cancer screening studies.

	Baseline round		Repeat rounds		Summary	
	Stage I (%) (95% CI)	*P*	Stage I (%) (95% CI)	*P*	Stage I (%) (95% CI)	*P*
Overall	70.1(63.9,76.0)	<0.001	75.6(70.0,80.9)	0.006	73.2(68.6,77.5)	<0.001
Region		0.203^a^		<0.001^a^		<0.001^a^
Asia	77.2(63.9,88.5)	0.026	90.2(82.3,96.4)	0.931	83.5(77.0,89.1)	0.133
Europe	69.8(60.0,78.9)	0.004	78.5(73.6,83.1)	0.953	73.0(68.2,77.5)	0.072
North America	63.7(56.3,70.7)	0.138	59.8(48.8,70.3)	0.145	63.0(57.7,68.1)	0.222
Study design		0.001^a^		0.524^a^		0.013^a^
cohort study	75.2(67.8,82.0)	0.001	78.0(70.2,85.1)	0.100	77.0(71.2,82.4)	<0.001
randomized controlled trial	59.8(55.1,64.5)	0.612	72.5(64.2,80.1)	0.015	66.8(61.3,72.1)	0.023
Initial screen year		0.274^a^		0.476‡		0.272^a^
before 2000	75.0(62.3,86.0)	<0.001	79.5(67.9,89.6)	0.021	76.9(67.2,85.4)	<0.001
after 2000	66.8(60.5,72.7)	0.012	72.5(66.9,77.8)	0.125	70.7(66.2,75.0)	0.002
Study quality		0.982^a^		0.039^a^		0.295^a^
high	68.8(59.7,77.3)	<0.001	71.3(62.8,79.2)	0.019	70.3(63.9,76.3)	<0.001
moderate	70.3(61.8,78.2)	0.084	81.3(75.9,86.2)	0.551	75.4(69.7,80.7)	0.010
low	69.8(57.4,81.0)	<0.001	60.4(33.4,85.1)	<0.001	67.6(56.7,77.7)	<0.001
Minimum age≥50 years old		0.391^a^		0.005^a^		0.057^a^
yes	67.8(61.6,73.7)	0.002	71.8(65.9,77.5)	0.024	69.8(65.3,74.1)	0.001
not restricted	76.0(59.1,89.9)	0.002	89.1(81.1,95.6)	0.810	80.2(70.9,88.3)	0.001
Smokers/ex-smokers		0.362^a^		0.015^a^		0.015^a^
yes	68.1(62.0,74.0)	0.001	73.8(68.0,79.2)	0.018	70.5(66.1,74.7)	<0.001
not restricted	77.1(58.3,91.9)	0.010	89.8(78.7,97.7)	0.722	83.5(74.1,91.2)	0.057

*P* value for comparison between subgroups denoted with ^a^, otherwise for the heterogeneity test.

Six studies [4–6,9,19,20] provided individual patient data that made more in-detail patient level subgroup analysis available (Table 3). There were decreasing trends of stage I cancer proportion with age and nodule diameter at the time of detection, from 85.9% for those aged <50 years to 74.2% for those aged ≥ 60 years, *P*_trend_ = 0.0509, and from 82.3% for nodules <10 mm to 62.4% for nodules ≥ 15mm, *P*_trend_ = 0.0120. Females (82.1%) showed a higher stage I cancer proportion than males (71.8%), but this difference was not statistically significant, *P =* 0.1795.

**Table 3:** Individual patient data subgroup analysis of sources of heterogeneity regarding the proportion of stage I cancers in CT lung cancer screening studies.

Category	Subgroup	n	Stage I (%) (95% CI)	*P* ^a^
Age at detection (year)	<50	18	85.9 (46.2,81.6)	0.0509
	50~	30	79.7 (55.7,80.1)	
	60~	91	74.2 (39.4,74.4)	
Diameter at detection (mm)	<10	53	82.3 (62.3,82.5)	0.0120
	10~	40	80.8 (50.0,81.1)	
	15~	46	62.4 (37.6,62.8)	
Gender	female	47	82.1 (54.0,82.4)	0.1795
	male	80	71.8 (43.5,72.0)	

Individual data extracted from Münster[4], Mobile[5], Israel[6], Samsung[9], ITALUNG[19], and DLCST[20] studies.

^a^ trend test for age and diameter, and chi-square test for gender difference

### Round specific changing trends over time in CT screens

**Fig 2:**
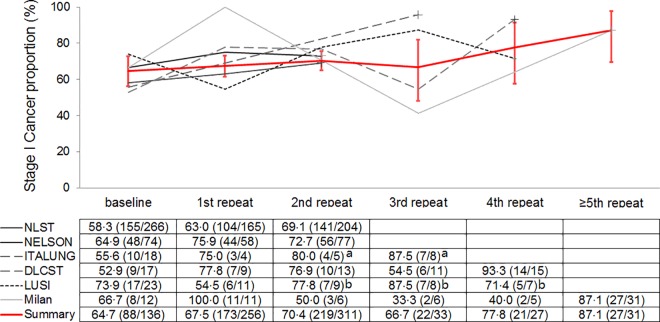
Proportion of round specific stage I cancer in six studies and summary estimates with 95% confident interval lines. Data are shown as stage I cancers/total cancers. Cancer stages and the detected rounds were determined by the time they were diagnosed. ^a^ and ^b^: separately summarized and smoothed in the figure lines because of the small total number of cancers (<9).

To provide round-specific changes as the screening programs progressed, we pooled the results from six studies [11,15,18–20,23] that provided detailed stage information for each screening round (Fig 2). Although the proportion of early cancers in single studies fluctuated over time, the pooled estimate showed an approximately perfect rising trend from baseline screening (64.7%) to the fifth and higher repeat rounds (87.1%; *P*_trend_<0.05). This rising trend seemed not to be explained by the influences of patient age or nodule size, as data extracted from the ITALUNG study [19] (the single study that reported such round-specific patient information, in which a rising stage I cancer proportion of 75.0–80.0–87.5% in the three repeat rounds was reported) showed no similar change in either average age (67.5–66.0–67.0 years) or average nodule diameter (8.3–10.6–8.4 mm), both *P*>0.05.

### Comparison between cancers detected using CT and other detection routes

**Fig 3:**
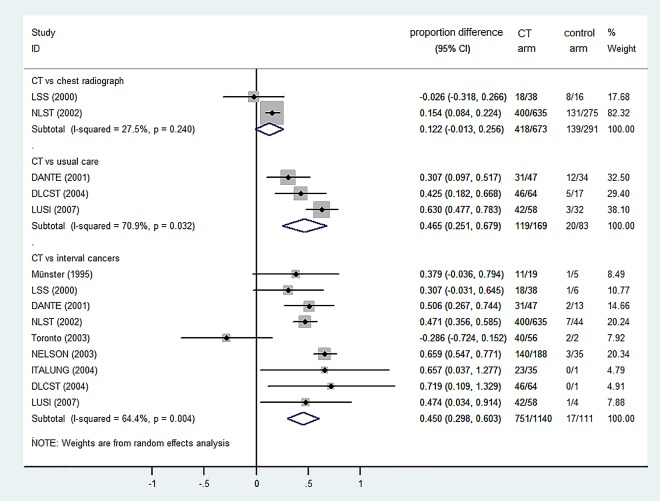
Differences between the proportion of stage I cancers detected using CT and other routes (data shown as CT minus other routes).

As compared with chest radiograph (Fig 3), the proportion of stage I lung cancer increased by 12.2% using CT, based on pooled results from NLST and its pilot trial (LSS) (*P*>0.05). When comparing results in the usual care arms, this advantage increased to 46.5% based on three European RCTs (*P*<0.05). For comparisons with interval cancers (diagnosed out of screening) using the available data from nine studies, the difference was almost identical (45.0%; *P*<0.05). The absolute difference in proportions can be translated into more than 2.5 fold differences in relative numbers if combined with the robust summary estimates using 24 studies, to be specific, 73.2% in CT vs 26.7% in usual care, and vs 28.2% of interval cancers. Still, analysis with the Egger’s test (not performed for the comparison with chest radiograph as there were only two studies) did not reveal any small-study bias, either in comparison with usual care (*P* = 0.200), or with interval cancers (*P* = 0.211).

### Pathological distribution and pathology-specific stages

**Fig 4:**
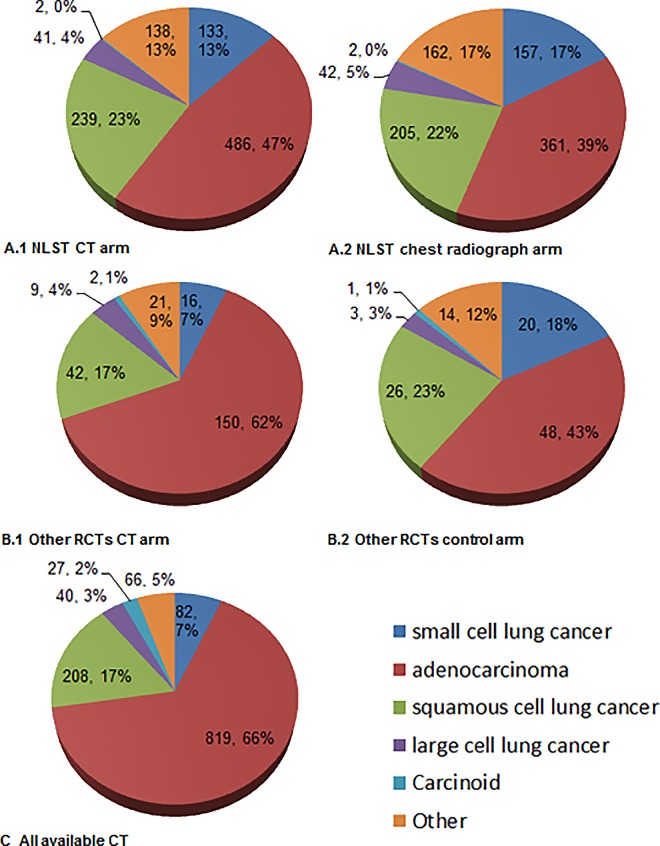
Lung cancer distributions according to the reported histological types. (A.1) Cancers detected in the CT arm of the National Lung Study Trial. (A.2) Cancers detected in the chest radiograph arm of the National Lung Study Trial. (B.1) Summary results for CT detected cancers from randomized control trials (RCTs) other than the National Lung Study Trial. (B.2) Summary results for cancers detected in the control arms (chest radiograph or usual care) from RCTs other than the National Lung Study Trial. (C) Summary results for CT detected cancers from all available reports (including cohorts and RCTs). Only studies reporting the full spectrum of histological compositions were used.

Adenocarcinoma was the most common lung cancer type (Fig 4), accounting for 39% of the cancers detected using chest radiograph in the NLST, and 43% in control arms in other RCTs (NLST data was separately shown because it was the most extensive and greatly influenced the pooled results). In the CT screens, these percentages increased significantly to 47% (NLST), 62% (other RCTs), and 66% (further added cohort studies) (all *P*<0.05 for comparison). Next was squamous cell lung cancer, whose absolute number increased when moving from the control arm to the CT arm in NLST and other RCTs, but the composition percentage fell because of the large increase in adenocarcinoma in RCTs other than the NLST. As to the most progressive type, namely small cell lung cancers, the composition percentages shrank in both the NLST and other RCTs using CT (17% vs 13% [*P*<0.05], and 18% vs 7% [*P*>0.05], respectively).

**Fig 5:**
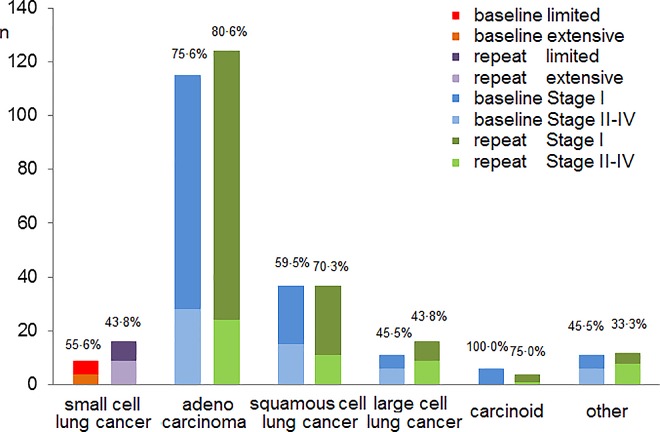
Pathologically specific stage distributions of lung cancers detected using CT in six studies. Data pooled from the Münster[4], Israel[6], Mayo[8], Toronto[16],NELSON[18],and ITALUNG[19]studies.

Pathology-specific differences in the proportion of early cancer between baseline and the repeat rounds are presented in Fig 5. Adenocarcinomas and squamous cell lung cancers predominated regarding the increase in the proportion of early cancer from baseline to the repeat rounds (75.6% to 80.6%, and 59.5% to 70.3%, respectively, both *P*>0.05), while in contrast the infrequent pathological types (e.g. limited stage proportion of small cell lung cancer from 55.6% to 43.8%) fell slightly in the repeat rounds (all *P*>0.05).

Data were only available from the NLST for comparison of the pathology-specific early stage cancer proportions between screening methods [15]. Significantly higher proportions of early stage adenocarcinomas (BACs: 80.9% vs 51.4%, *P =* 0.0006; other adenocarcinomas: 58.8% vs 38.3%, *P*<0.0001), squamous cell lung cancers (52.3% vs 39.0%, *P =* 0.0051) and some other non-small cell carcinomas were detected using CT than using chest radiograph (other non-small cell lung cancers combined: 40.1% vs 21.8%, *P*<0.0001); however, there was no such significant “shift” regarding the detection of early stage small cell lung cancers (6.8% vs 10.8%, *P =* 0.2277).

## Discussion

For over half a century, attempts to reduce lung cancer mortality using screening modalities were based on the assumption that early detection and treatment of malignant pulmonary nodules leads to improved prognosis [2]. A number of reviews are now available regarding the summary of current knowledge on the mortality outcome in CT screening [38,39]. Because of the paucity of outcome evidence and difficulties in uniformly measuring treatment effectiveness, the present study focused on the topic of the first “detection” step and provided several deep insights regarding lung cancer stage. The results showed that >70% of non-small cell lung cancer patients detected using CT were at pathological stage I, and there is a tendency for this proportion to increase as screening continues. Relative to chest radiograph screening and usual care, the proportion of stage I cancer detected using CT was higher by more than 12% and 45%, respectively. Regarding pathology, almost all types of non-small cell lung cancer can increasingly be detected at an early stage in a CT rather than in a chest radiograph screening; however, evidence is lacking regarding this advantage in small cell lung cancer patients.

As can also be inferred from the current study, different populations and study designs can to a great extent affect the stage characteristics of the detected lung cancers. Relative to patients in North America and Europe, the proportion of Stage I cancer was higher in Asian patients (83.5% in baseline and repeat summary), with three Japanese studies dominating the results. This could possibly be explained by the long history of lung screening in Japan [40], whose high proportion of early stage cancer observed complied well with the trend of long-term repeat screens (Fig 2). Studies that were restricted to populations aged ≥50 years or smokers/ex-smokers had lower proportions of early stage cancer, potentially indicating a high proportion of late stage cancers in these subgroups; this is supported by the individual patient data subgroup analysis on age in this study, and the finding from three Chinese cities with a high lung cancer incidence (stage I cancer accounted for as low as 34.3% of the CT detected lung cancers) [41]. In addition, differences in study designs and study qualities reflect variations in modality parameters, threshold for follow-up, scan intervals (ranging from twice a year in the ALCAP to annual/biennial in the MILD, and a 1-3-5.5 year scheme in the NELSON trial) [3,18,22], and some other aspects of varied protocols that could have an impact on detection performance. All of these differences in screening yields stress the necessity that a cancer screening service should be delivered in a culturally sensitive and methodologically sound manner to optimize public health benefits [26].

Notably, the changing pattern of early cancer proportion also holds some implications for screening practice. Although the absolute number of lung cancers detected decreased in the repeat round relative to the initial screening (the case in almost all of the included studies), the proportion of early cancers could rise as screening continues. This is especially true when the ideal status is reached where only the most recently developed cancers are left to be detected in long-term screening (e.g., approaching 90% in the fifth and higher repeat rounds; however, it should be noted that this estimate came from just one study). Thus, it is suggested that the “detect early to increase curability” vision can only be more ideally fulfilled when screening is continued over time, rather than as a one-off practice, which is effectively no more than a “prevalence survey”.

Currently there are two opinions on the observed “stage shift” in CT screening as compared with other detection routes (e.g., stage I cancer proportion increased by 46.5% than in usual care in this study, representing a more than 2.5 fold difference). Traditional lung cancer natural history theory holds that screening reveals many of the cancers that can be treated at an early stage before they become incurable (stage shift) [42]. However, the alternative opinion is not that optimistic, doubting that historically indolent cancers dominate the majority of “early detected” cancers (i.e. “histology shift” instead of real stage shift) [43], thus weakening the effectiveness of screening and resulting in unnecessary diagnosis and even over-treatment [32,43,44]. Therefore, analyses involving pathology could provide some insightful information regarding the discrepancy in “detecting cancer at an early stage” and “detecting early cancers” [45]. Veronesi et al reported that slow-growing cancer comprised about 25% of the CT detected lung cancers [46], and several similar studies found about 80% of such indolent cancers were BAC or adenocarcinomas [43,46,47]. In the study by Vazquez et al, the majority (95%) of adenocarcinomas had a BAC component, and the 10-year survival rates after resection were as high as 90%-100% [48]. It is also indicated that an increase in the recorded incidence of BAC in the past 30 years was partly attributable to CT scanning [49]. In an NLST subgroup, Young et al further revealed that BAC cancers were preferentially identified by CT screening and almost exclusively found in those with no airflow limitation [43]. In our pooled study, about 18.6% of adenocarcinomas (the most common type of cancer detected in CT screening, 66% of all cancers) were classified as BAC (consistent with a review article) [49]. All these strongly suggest that indolent cancers such as BAC could have contributed an unelectable portion of the additionally detected cancers. With prostate and breast cancer screening as predecessors, caution should be taken when interpreting the impressive “detection improvement” statistics [50]. In addition, the need should be stressed to separately report BAC from other adenocarcinomas (for example, the separated analyses were not performed in Figs 4 and 5 because nine studies did not make a difference between BAC and other adenocarcinomas), or to report according to the new classification system [36] the subtypes of cancers that have a favorable prognosis and are traditionally diagnosed as BAC (such as adenocarcinoma in situ, minimally invasive adenocarcinoma, and lepidic-predominant adenocarcinoma), because clumping them into overall adenocarcinoma will mean their relevance to true efficiency in screening programs may be lost.

In contrast to the non-small cell lung cancers, there is currently no evidence showing the superiority of early detection using CT for the more aggressive small cell lung cancer, and the most recent report from the MILD study showed that CT did not improve survival for such cancer types (no small cell lung cancer survivors after 3 years) [51]. The marked difference of early cancer detectability in CT screening for indolent and aggressive cancers could possibly provide one explanation regarding the phenomenon that early detection does not always relate to decreased mortality in small RCTs [19,20,22,23]. We noticed that one study named lungSEARCh that aimed to demonstrate a stage shift towards early stage cancers have just finished its follow-up by March 2016 [52], and hope it could provide more histology-specific insights into such difference. Further, as volume doubling time can serve as a good way to monitor cancer behaviors [18,46], more studies on the heterogeneity of volume doubling time for different histological types of lung cancers (especially the non-small cell ones) are warranted for better defining cancer biology, predicting screening outcomes, and refining screening protocols.

Limitations remain in this study. First, though random effect model was applied to obtain conservative pooled results of the early stage lung cancer proportion, the averaged estimates could not cover all possibilities of CT screening findings as the patients backgrounds, CT techniques, and screening workflows (impractical to be categorized for analysis) in this study were quite various among all studies, especially when considering the diversity of cancers with different biological behaviors and future development in screening techniques. Second, sources of heterogeneity were not fully explored, as indicated by the heterogeneities persisted within most small subgroups in the study level analysis; the individual patient level data analysis can provide a way for more insights with greater accuracy, but it was limited by the small number of available subjects. Third, though no small study/publication bias was detected, as all the included studies were in English and Chinese, language bias may exist. Last, due to lack of sufficient evidence in the ultimate outcome of screening program (lung cancer mortality), and due to the inconsistency in the methods to determine over-diagnosis [39], it is still impractical to directly estimate the degree of correlation between “stage shift” and mortality reduction.

In conclusion, CT has superiority over chest radiograph and usual care for detecting a higher proportion of early stage non-small cell lung cancers, including a number of indolent cancers such as BAC. However, evidence is currently lacking for the same beneficial stage shift of the more aggressive small cell lung cancers.

## Supporting Information

S1 Table PRISMA checklist. (DOCX)

S2 Table Extracted data used in this meta-analysis. (XLSX)

S3 Table Domains and index questions for study quality assessment. (DOCX)

S4 Table Study quality assessment results. (DOCX)

S1 Text Electronic search strategy record. (DOCX)

S2 Text List of the full-text excluded articles with reasons. (DOCX)

## References

[1] International Agency for Research on Cancer. GLOBOCAN 2012: Estimated Cancer Incidence, Mortality and Prevalence Worldwide in 2012. Available: http://globocan.iarc.fr/Pages/fact_sheets_cancer.aspx.

[2] Manser R, Lethaby A, Irving LB, Stone C, Byrnes G, Abramson MJ, et al Screening for lung cancer. Cochrane Database Syst Rev. 2013;6: CD001991 10.1002/14651858.CD001991.pub3 23794187 PMC6464996

[3] Seki N, Eguchi K, Kaneko M, Ohmatsu H, Kakinuma R, Matsui E, et al The adenocarcinoma-specific stage shift in the Anti-lung Cancer Association project: significance of repeated screening for lung cancer for more than 5 years with low-dose helical computed tomography in a high-risk cohort. Lung Cancer. 2010;67: 318–324. 10.1016/j.lungcan.2009.04.016 19481832

[4] Diederich S, Thomas M, Semik M, Lenzen H, Roos N, Weber A, et al Screening for early lung cancer with low-dose spiral computed tomography: results of annual follow-up examinations in asymptomatic smokers. Eur Radiol. 2004;14: 691–702. 10.1007/s00330-003-2200-5 14727146

[5] Li F, Sone S, Abe H, MacMahon H, Doi K. Low-dose computed tomography screening for lung cancer in a general population: characteristics of cancer in non-smokers versus smokers. Acad Radiol. 2003;10: 1013–1020. 13678090 10.1016/s1076-6332(03)00150-8

[6] Shaham D, Breuer R, Copel L, Agid R, Makori A, Kisselgoff D, et al Computed tomography screening for lung cancer: applicability of an international protocol in a single-institution environment. Clin Lung Cancer. 2006;7: 262–267. 10.3816/CLC.2006.n.004 16512980

[7] Nawa T, Nakagawa T, Kusano S, Kawasaki Y, Sugawara Y, Nakata H. Lung cancer screening using low-dose spiral CT: results of baseline and 1-year follow-up studies. Chest. 2002;122: 15–20. 12114333 10.1378/chest.122.1.15

[8] Swensen SJ, Jett JR, Hartman TE, Midthun DE, Mandrekar SJ, Hillman SL, et al CT screening for lung cancer: five-year prospective experience. Radiology. 2005;235: 259–265. 10.1148/radiol.2351041662 15695622

[9] Chong S, Lee KS, Chung MJ, Kim TS, Kim H, Kwon OJ, et al Lung cancer screening with low-dose helical CT in Korea: experiences at the Samsung Medical Center. J Korean Med Sci. 2005;20: 402–408. 10.3346/jkms.2005.20.3.402 15953860 PMC2782194

[10] Bastarrika G, García-Velloso MJ, Lozano MD, Montes U, Torre W, Spiteri N, et al Early lung cancer detection using spiral computed tomography and positron emission tomography. Am J Respir Crit Care Med. 2005;171: 1378–1383. 10.1164/rccm.200411-1479OC 15790860

[11] Veronesi G, Maisonneuve P, Rampinelli C, Bertolotti R, Petrella F, Spaggiari L, et al Computed tomography screening for lung cancer: results of ten years of annual screening and validation of cosmos prediction model. Lung Cancer. 2013; 82: 426–430. 10.1016/j.lungcan.2013.08.026 24099665

[12] Gohagan JK, Marcus PM, Fagerstrom RM, Pinsky PF, Kramer BS, Prorok PC, et al Final results of the Lung Screening Study, a randomized feasibility study of spiral CT versus chest X-ray screening for lung cancer. Lung Cancer. 2005; 47:9–15. 10.1016/j.lungcan.2004.06.007 15603850

[13] Infante M, Cavuto S, Lutman FR, Brambilla G, Chiesa G, Ceresoli G, et al A randomized study of lung cancer screening with spiral computed tomography: three-year results from the DANTE trial. Am J RespirCrit Care Med. 2009;180: 445–453. 10.1164/rccm.200901-0076OC 19520905

[14] Wilson DO, Weissfeld JL, Fuhrman CR, Fisher SN, Balogh P, Landreneau RJ, et al The Pittsburgh Lung Screening Study (PLuSS): outcomes within 3 years of a first computed tomography scan. Am J RespirCrit Care Med. 2008;178: 956–961. 10.1164/rccm.200802-336OC PMC2720144 18635890

[15] National Lung Screening Trial Research Team, Aberle DR, Adams AM, Berg CD, Black WC, Clapp JD, et al Reduced lung-cancer mortality with low-dose computed tomographic screening. N Engl J Med. 2011;365: 395–409. 10.1056/NEJMoa1102873 21714641 PMC4356534

[16] Menezes RJ, Roberts HC, Paul NS, McGregor M, Chung TB, Patsios D, et al Lung cancer screening using low-dose computed tomography in at-risk individuals: the Toronto experience. Lung Cancer. 2010;67: 177–183. 10.1016/j.lungcan.2009.03.030 19427055

[17] Liu X, Liang M, Wang Y, Chen K, Chen X, Qin P, et al The outcome differences of CT screening for lung cancer pre and post following an algorithm in Zhuhai, China. Lung Cancer. 2011;73: 230–236. 10.1016/j.lungcan.2010.11.012 21168238

[18] Horeweg N, Scholten ET, de Jong PA, van der Aalst CM, Weenink C, Lammers JW, et al Detection of lung cancer through low-dose CT screening (NELSON): a prespecified analysis of screening test performance and interval cancers. Lancet Oncol. 2014;15: 1342–1350. 10.1016/S1470-2045(14)70387-0 25282284

[19] Lopes Pegna A, Picozzi G, Falaschi F, Carrozzi L, Falchini M, Carozzi FM, et al Four-year results of low-dose CT screening and nodule management in the ITALUNG trial. J Thorac Oncol. 2013;8: 866–875. 10.1097/JTO.0b013e31828f68d6 23612465

[20] Saghir Z, Dirksen A, Ashraf H, Bach KS, Brodersen J, Clementsen PF, et al CT screening for lung cancer brings forward early disease. The randomised Danish Lung Cancer Screening Trial: status after five annual screening rounds with low-dose CT. Thorax. 2012;67: 296–301. 10.1136/thoraxjnl-2011-200736 22286927

[21] Pelosi G, Sonzogni A, Veronesi G, De Camilli E, Maisonneuve P, Spaggiari L, et al Pathologic and molecular features of screening low-dose computed tomography (LDCT)-detected lung cancer: a baseline and 2-year repeat study. Lung Cancer. 2008;62: 202–214. 10.1016/j.lungcan.2008.03.012 18450320

[22] Pastorino U, Rossi M, Rosato V, Marchianò A, Sverzellati N, Morosi C, et al Annual or biennial CT screening versus observation in heavy smokers: 5-year results of the MILD trial. Eur J Cancer Prev. 2012;21: 308–315. 10.1097/CEJ.0b013e328351e1b6 22465911

[23] Becker N, Motsch E, Gross ML, Eigentopf A, Heussel CP, Dienemann H, et al Randomized Study on Early Detection of Lung Cancer with MSCT in Germany: Results of the First 3 Years of Follow-up After Randomization. J Thorac Oncol. 2015;10: 890–896. 10.1097/JTO.0000000000000530 25783198

[24] Tang W, Wu N, Huang Y, Wang J, Zhao S, Xu Z, et al Results of low-dose computed tomography (LDCT) screening for early lung cancer: prevalence in 4690 asymptomatic participants. ZhonghuaZhong Liu ZaZhi. 2014;36: 549–554.[in Chinese] 25327664

[25] Rzyman W, Dziedzic R, Jelitto-Górska M, Biadacz I, Książek J, Siebert J, et al Results of an open-access lung cancer screening program with low-dose computed tomography: the Gdańsk experience. Pol Arch Med Wewn. 2015;125: 232–239. 25764248 10.20452/pamw.2778

[26] McKee BJ, Hashim JA, French RJ, McKee AB, Hesketh PJ, Lamb CR, Williamson C, et al Experience with a CT screening program for individuals at high risk for developing lung cancer. J Am Coll Radiol. 2015;12: 192–197. 10.1016/j.jacr.2014.08.002 25176498

[27] Mulshine JL, D'Amico TA. Issues with implementing a high-quality lung cancer screening program. CA Cancer J Clin. 2014;64: 351–363. 10.3322/caac.21239 24976072

[28] Jones GS, Baldwin DR. Lung cancer screening and management. Minerva Med. 2015;106: 339–354. 26605556

[29] Seki N, Eguchi K, Kaneko M, Ohmatsu H, Kakinuma R, Matsui E, et al The adenocarcinoma-specific stage shift in the Anti-lung Cancer Association project: significance of repeated screening for lung cancer for more than 5 years with low-dose helical computed tomography in a high-risk cohort. Lung Cancer. 2010;67: 318–324. 10.1016/j.lungcan.2009.04.016 19481832

[30] Bach PB. Is our natural-history model of lung cancer wrong? Lancet Oncol. 2008;9: 693–697. 10.1016/S1470-2045(08)70176-1 18598934

[31] Hillerdal G. Indolent lung cancers—time for a paradigm shift: a review. J Thorac Oncol. 2008;3: 208–211. 10.1097/JTO.0b013e3181653ce3 18317061

[32] Patz EF Jr, Pinsky P, Gatsonis C, Sicks JD, Kramer BS, Tammemägi MC, et al Overdiagnosis in low-dose computed tomography screening for lung cancer. JAMA Intern Med. 2014;174: 269–274. 10.1001/jamainternmed.2013.12738 24322569 PMC4040004

[33] Young RP, Hopkins RJ. Mortality Reduction, Overdiagnosis, and the Benefit-to-Harm Ratio of Computed Tomography Screening. Am J Respir Crit Care Med. 2015;192: 398–399. 10.1164/rccm.201504-0801LE 26230243

[34] Whiting PF, Rutjes AW, Westwood ME, Mallett S, Deeks JJ, Reitsma JB, et al QUADAS-2: a revised tool for the quality assessment of diagnostic accuracy studies. Ann Intern Med. 2011;155: 529–536. 10.7326/0003-4819-155-8-201110180-00009 22007046

[35] Edge SB, Compton CC. The American Joint Committee on Cancer: the 7th Edition of the AJCC Cancer Staging Manual and the Future of TNM. Ann Surg Oncol. 2010;17: 1471–1474. 10.1245/s10434-010-0985-4 20180029

[36] Travis WD, Brambilla E, Noguchi M, Geisinger K, Beer D, Powell CA, et al The new IASLC/ATS/ERS international multidisciplinary lung adenocarcinoma classification. J Thorac Oncol. 2011;6: 244–285. 10.1097/JTO.0b013e318206a221 21252716 PMC4513953

[37] Nyaga VN, Arbyn M, Aerts M. Metaprop: a Stata command to perform meta-analysis of binomial data. Arch Public Health. 2014;72: 39 10.1186/2049-3258-72-39 25810908 PMC4373114

[38] Humphrey LL, Deffebach M, Pappas M, Baumann C, Artis K, Mitchell JP, et al Screening for lung cancer with low-dose computed tomography: a systematic review to update the US Preventive services task force recommendation. Ann Intern Med. 2013;159: 411–420. 10.7326/0003-4819-159-6-201309170-00690 23897166

[39] Usman Ali M, Miller J, Peirson L, Fitzpatrick-Lewis D, Kenny M, Sherifali D, et al Screening for lung cancer: A systematic review and meta-analysis. Prev Med. 2016;89: 301–314. 10.1016/j.ypmed.2016.04.015 27130532

[40] Nawa T, Nakagawa T, Mizoue T, Endo K. Low-dose computed tomography screening in Japan. J Thorac Imaging. 2015;30: 108–114. 10.1097/RTI.0000000000000138 25658475

[41] Zhao SJ, Wu N. Early detection of lung cancer: Low-dose computed tomography screening in China. Thorac Cancer. 2015;6: 385–389. 10.1111/1759-7714.12253 26273391 PMC4511314

[42] Henschke CI, Yankelevitz DF, Libby D, Kimmel M. CT screening for lung cancer: the first ten years. Cancer J. 2002;suppl1: S47–S54. 12075702

[43] Young RP, Duan F, Chiles C, Hopkins RJ, Gamble GD, Greco EM, et al Airflow Limitation and Histology Shift in the National Lung Screening Trial. The NLST-ACRIN Cohort Substudy. Am J Respir Crit Care Med. 2015;192: 1060–1067. 10.1164/rccm.201505-0894OC 26199983 PMC4642202

[44] Braillon A. Bronchioalveolar lung cancer: screening and overdiagnosis. J Clin Oncol. 2014;32: 3575. 10.1200/JCO.2014.55.9732 25225426

[45] Young RP, Hopkins RJ. Stage shift in computed tomography screening: possible role of indolent cancers, "histology shift," and overdiagnosis. Am J Respir Crit Care Med. 2013;188: 1034–1035. 10.1164/rccm.201305-0832LE 24127805

[46] Veronesi G, Maisonneuve P, Bellomi M, Rampinelli C, Durli I, Bertolotti R, et al Estimating overdiagnosis in low-dose computed tomography screening for lung cancer: a cohort study. Ann Intern Med. 2012;157: 776–784. 10.7326/0003-4819-157-11-201212040-00005 23208167

[47] Wilson DO, Ryan A, Fuhrman C, Schuchert M, Shapiro S, Siegfried JM, et al Doubling times and CT screen–detected lung cancers in the Pittsburgh Lung Screening Study. Am J Respir Crit Care Med. 2012;185: 85–89. 10.1164/rccm.201107-1223OC 21997335 PMC3262038

[48] Vazquez M, Carter D, Brambilla E, Gazdar A, Noguchi M, Travis WD, et al Solitary and multiple resected adenocarcinomas after CT screening for lung cancer: histopathologic features and their prognostic implications. Lung Cancer. 2009;64: 148–154. 10.1016/j.lungcan.2008.08.009 18951650 PMC2849638

[49] Yousem SA, Beasley MB. Bronchioloalveolar Carcinoma: A Review of Current Concepts and Evolving Issues. Arch Pathol Lab Med. 2007;131: 1027–1032. 10.1043/1543-2165(2007)131[1027:BCAROC]2.0.CO;2 17616987

[50] Silvestri GA. Screening for lung cancer: it works, but does it really work? Ann Intern Med. 2011;155: 537–539. 10.7326/0003-4819-155-8-201110180-00364 21893614

[51] Silva M, Galeone C, Sverzellati N, Marchianò A, Calareso G, Sestini S, et al Screening with Low-Dose Computed Tomography Does Not Improve Survival of Small Cell Lung Cancer. J Thorac Oncol. 2016;11: 187–193. 10.1016/j.jtho.2015.10.014 26845115

[52] Spiro SG, Hackshaw A, LungSEARCH Collaborative Group. Research in progress-LungSEARCH: a randomised controlled trial of surveillance for the early detection of lung cancer in a high-risk group. Thorax. 2016;71: 91–93. 10.1136/thoraxjnl-2015-207433 26138736 PMC4717418

