# Supplementary material for: Can CT Screening Give Rise to a Beneficial Stage Shift in Lung Cancer Patients? Systematic Review and Meta-Analysis
Source: PLoS One. 2016 Oct 13;11(10):e0164416. doi: 10.1371/journal.pone.0164416 (PMC5063401; doi:10.1371/journal.pone.0164416)
Supplement: S3 Table — (DOCX) [file pone.0164416.s003.docx]

| **S3 Table. Domains and index questions for study quality assessment** | | |
| --- | --- | --- |
| **Domain** | **Mark** | **Question** |
| PATIENT SELECTION | R1.1 | Was a consecutive or random sample of patients enrolled? |
|  | R1.2 | Did the study avoid inappropriate exclusions? |
|  | C1 | Is there concern that the included patients do not match the review question? |
|  |  |  |
| INDEX TEST | R2.1 | Were the index test results interpreted without knowledge of the results of the reference standard? |
|  | R2.2 | If a threshold was used, was it pre-specified? |
|  | C2 | Is there concern that the index test, its conduct, or interpretation differ from the review question? |
|  |  |  |
| REFERENCE STANDARD | R3.1 | Is the reference standard likely to correctly classify the target condition? |
|  | R3.2 | Were the reference standard results interpreted without knowledge of the results of the index test? |
|  | C3 | Is there concern that the target condition as defined by the reference standard does not match the review question? |
|  |  |  |
| FLOW AND TIMING | R4.1 | Was there an appropriate interval between index test(s) and reference standard? |
|  | R4.2 | Did all patients receive the same reference standard? |
|  | R4.3 | Were all patients included in the analysis? |
| R: risk of bias C: concern of applicability. | | |
