# Supplementary material for: Can CT Screening Give Rise to a Beneficial Stage Shift in Lung Cancer Patients? Systematic Review and Meta-Analysis
Source: PLoS One. 2016 Oct 13;11(10):e0164416. doi: 10.1371/journal.pone.0164416 (PMC5063401; doi:10.1371/journal.pone.0164416)
Supplement: S4 Table — (DOCX) [file pone.0164416.s004.docx]

| **S4 Table. Study quality assessment results** | | | | | | | | | | | | | |
| --- | --- | --- | --- | --- | --- | --- | --- | --- | --- | --- | --- | --- | --- |
| **Study** | **PATIENT SELECTION** | | | **INDEX TEST** | | | **REFERENCE STANDARD** | | | **FLOW AND TIMING** | | | **Rank** |
|  | R1.1 | R1.2 | C1 | R2.1 | R2.2 | C2 | R3.1 | R3.2 | C3 | R4.1 | R4.2 | R4.3 |  |
| ALCAP[3] | 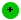 | 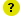 | 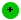 | 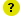 | 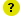 | 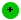 | 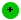 | 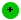 | 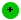 | 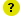 | 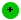 | 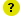 | moderate |
| Münster[4] | 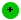 | 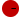 | 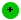 | 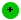 | 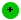 | 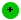 | 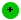 | 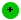 | 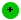 | 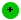 | 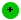 | 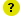 | moderate |
| Mobile[5] | 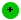 | 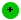 | 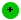 | 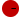 | 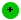 | 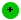 | 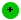 | 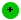 | 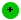 | 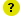 | 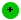 | 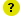 | moderate |
| Israel[6] | 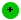 | 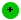 | 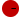 | 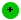 | 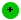 | 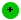 | 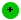 | 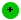 | 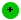 | 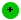 | 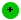 | 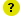 | moderate |
| Hitachi[7] | 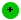 | 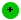 | 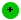 | 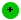 | 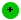 | 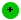 | 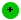 | 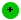 | 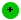 | 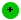 | 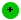 | 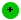 | high |
| Mayo[8] | 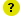 | 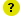 | 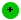 | 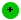 | 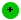 | 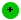 | 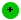 | 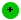 | 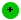 | 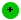 | 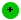 | 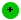 | high |
| Samsung[9] | 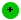 | 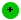 | 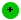 | 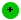 | 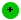 | 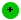 | 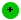 | 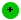 | 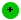 | 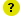 | 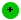 | 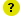 | high |
| Spain[10] | 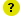 | 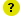 | 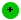 | 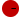 | 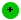 | 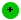 | 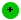 | 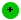 | 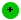 | 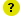 | 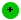 | 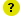 | low |
| Milan[11] | 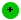 | 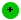 | 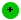 | 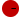 |  |  |  |  |  |  |  |  | moderate |
| LSS[12] |  |  |  |  |  |  |  |  |  |  |  |  | high |
| DANTE[13] |  |  |  |  |  |  |  |  |  |  |  |  | high |
| PLuSS[14] |  |  |  |  |  |  |  |  |  |  |  |  | low |
| NLST[15] |  |  |  |  |  |  |  |  |  |  |  |  | high |
| Toronto[16] |  |  |  |  |  |  |  |  |  |  |  |  | moderate |
| Zhuhai[17] |  |  |  |  |  |  |  |  |  |  |  |  | moderate |
| NELSON[18] |  |  |  |  |  |  |  |  |  |  |  |  | high |
| ITALUNG[19] |  |  |  |  |  |  |  |  |  |  |  |  | moderate |
| DLCST[20] |  |  |  |  |  |  |  |  |  |  |  |  | moderate |
| COSMOS[21] |  |  |  |  |  |  |  |  |  |  |  |  | moderate |
| MILD[22] |  |  |  |  |  |  |  |  |  |  |  |  | moderate |
| LUSI[23] |  |  |  |  |  |  |  |  |  |  |  |  | high |
| CAMS-h[24] |  |  |  |  |  |  |  |  |  |  |  |  | high |
| PLCSP[25] |  |  |  |  |  |  |  |  |  |  |  |  | high |
| Massachusetts[26] |  |  |  |  |  |  |  |  |  |  |  |  | moderate |

low risk of bias or concern of applicability;high risk of bias or concern of applicability;unclear.
