# Supplementary material for: Can CT Screening Give Rise to a Beneficial Stage Shift in Lung Cancer Patients? Systematic Review and Meta-Analysis
Source: PLoS One. 2016 Oct 13;11(10):e0164416. doi: 10.1371/journal.pone.0164416 (PMC5063401; doi:10.1371/journal.pone.0164416)
Supplement: S1 Text — (DOCX) [file pone.0164416.s005.docx]

**S1 Text. Electronic search strategy record**

*Date*: 26 Jul 2015

*Database*：EMBASE，MEDLINE

*Platform*: <http://www.embase.com/#advancedSearch/default>

*Retrieval formula*：

(‘CT’:ab OR ‘CT’:ti OR ‘Computed Tomography’:ab OR ‘Computed Tomography’:ti OR ‘Computerized Tomography’:ab OR ‘Computerized Tomography’:ti OR ‘XrayComputed’:ab OR ‘Xray Computed’:ti OR ‘Computed X-Ray’:ab OR ‘Computed X-Ray’:ti OR ‘CAT Scan’:ab OR ‘CAT Scan’:ti OR ‘CAT Scans’:ab OR ‘CAT Scans’:ti) AND (lung:ab OR lung:ti OR pulmonary:ab OR pulmonary:ti) AND (cancer:ab OR cancer:ti OR cancers:ab OR cancers:ti OR neoplasm:ab OR neoplasm:ti OR neoplasms:ab OR neoplasms:ti OR carcinoma:ab OR carcinoma:ti OR carcinomas:ab OR carcinomas:ti OR nodule:ab OR nodule:ti OR nodules:ab OR nodules:ti) AND (screening:ab OR screening:ti OR screenings:ab OR screenings:ti)

*Mapping options*：

Map to preferred term in Emtree

Search also as free text in all fields

Explode using narrower Emtree terms

Search as broadly as possible

*Date limits*: Jan 1 1990 to 26 Jul 2015

No any *other limits*
