# Supplementary material for: Can CT Screening Give Rise to a Beneficial Stage Shift in Lung Cancer Patients? Systematic Review and Meta-Analysis
Source: PLoS One. 2016 Oct 13;11(10):e0164416. doi: 10.1371/journal.pone.0164416 (PMC5063401; doi:10.1371/journal.pone.0164416)
Supplement: S2 Text — (DOCX) [file pone.0164416.s006.docx]

**S2 Text. List of the full-text excluded articles with reasons**

Fail to extract stage information (n=65)

[1] Zhang CF, Zeng Q, Wang WM, Feng D, He Y. Detection rates and cost of lung cancer screening with low-dose helical computed tomography among physical examination people. Chinese Journal of Cancer Prevention and Treatment. 2015;22:247-51.

[2] Yi CA, Lee KS, Shin MH, Cho YY, Choi YH, Kwon OJ, et al. Low-dose CT screening in an Asian population with diverse risk for lung cancer: A retrospective cohort study. European Radiology. 2015.

[3] Percac-Lima S, Ashburner JM, Rimmelin D, Atlas SJ. Follow-up of abnormal chest CT in smokers at high risk for developing lung cancer. Journal of General Internal Medicine. 2015;30:S164-S5.

[4] Henschke CI, Boffetta P, Yankelevitz DF, Altorki N. Computed Tomography Screening : The International Early Lung Cancer Action Program Experience. Thoracic Surgery Clinics. 2015;25:129-43.

[5] Zhou Q, Fan Y, Wu N, Huang Y, Wang Y, Li L, et al. Demonstration program of population-based lung cancer screening in China: Rationale and study design. Thoracic Cancer. 2014;5:197-203.

[6] Zhao YR, Heuvelmans MA, Dorrius MD, Van Ooijen PMA, Wang Y, De Bock GH, et al. Features of resolving and nonresolving indeterminate pulmonary nodules at follow-up CT: The NELSON study. Radiology. 2014;270:872-9.

[7] McKee BJ, Hashim JA, French RJ, McKee AB, Hesketh PJ, Lamb CR, et al. Experience With a CT Screening Program for Individuals at High Risk for Developing Lung Cancer. Journal of the American College of Radiology. 2014.

[8] Guldbrt LM, Rasmussen TR, Rasmussen F, Vedsted P. Implementing direct access to low-dose computed tomography in general practice method, adaption - And outcome. PLoS ONE. 2014;9.

[9] Goulart B, Madtes D, Koepl L, Karnopp A, Nelson JA, Ramsey SD. Performance of a low-dose computed tomography (LDCT) lung cancer screening program: Findings from a registry study. Journal of Clinical Oncology. 2014;32.

[10] Field JK, Devaraj A, Baldwin DR, Holemans J, Screaton N, Ledson M, et al. UK Lung Cancer Screening trial (UKLS): Prevalence data at baseline. Lung Cancer. 2014;83:S24-S5.

[11] Yi CA, Lee KS, Shin MH, Cho YY, Shin KE, Choi YH. Low-dose computed tomographic screening in healthy adults at low risk for lung cancer. Journal of Thoracic Oncology. 2013;8:S686-S7.

[12] McWilliams A, Tammemagi MC, Mayo JR, Roberts H, Liu G, Soghrati K, et al. Probability of cancer in pulmonary nodules detected on first screening CT. New England Journal of Medicine. 2013;369:910-9.

[13] Kakinuma R, Kusumoto M, Asamura H, Tsuta K, Tsuchida T, Tamura T, et al. Lung cancers detected using lowdose CT screening: Results of an eight-year observational study. Journal of Thoracic Oncology. 2013;8:S684.

[14] Juntima-Euathrongchit A, Kattipathanapong T, Wannasopha Y, Ua-Apisitwong S, Jirapong K, Saeteng S, et al. Comparison between chest radiography, chest tomosynthesis and computed tomography to detect pulmonary nodules: A phantom study and clinical experience. Journal of Thoracic Oncology. 2013;8:S685.

[15] Jin GY, Lynch D, Chawla A, Garg K, Tammemagi MC, Sahin H, et al. Interstitial lung abnormalities in a CT lung cancer screening population : Prevalence and progression rate. Radiology. 2013;268:563-71.

[16] Field JK, Devaraj A, Baldwin DR, Holemans J, Screaton N, Ledson M, et al. UK lung cancer screening trial (UKLS): Base line data. Journal of Thoracic Oncology. 2013;8:S685.

[17] Field JK, Baldwin D, Devaraj A, Brain K, Eisen T, Holemans J, et al. United Kingdom lung cancer screening trial (UKLS): First 88897 approaches. Cancer Research. 2013;73.

[18] Wilson DO, Ryan A, Fuhrman C, Schuchert M, Shapiro S, Siegfried JM, et al. Doubling times and CT screen-detected lung cancers in the Pittsburgh lung screening study. American Journal of Respiratory and Critical Care Medicine. 2012;185:85-9.

[19] Paulon E, O'Riordan D, Shenoy A. Incidence and management of malignant polyps in the North Essex bowel cancer screening programme. Gut. 2012;61:A329.

[20] McKee AB, McKee BJ, Hesketh PJ, Wald C, French RJ, Flacke S. Initial results of a nccn guideline-based free low-dose ct lung cancer screening program. International Journal of Radiation Oncology Biology Physics. 2012;84:S585.

[21] Marshall HM, Bowman RV, Crossin J, Lau MA, Slaughter RE, Ayres J, et al. The Queensland lung cancer screening study: Risk stratification using participant data and lung function tests can significantly increase screening effectiveness. Journal of Thoracic Oncology. 2012;7:S177.

[22] Marshall H. Low dose ct screening for lung cancer. Asia-Pacific Journal of Clinical Oncology. 2012;8:158.

[23] Kanashiki M, Tomizawa T, Yamaguchi I, Kurishima K, Hizawa N, Ishikawa H, et al. Volume doubling time of lung cancers detected in a chest radiograph mass screening program: Comparison with CT screening. Oncology Letters. 2012;4:513-6.

[24] Henschke CI, Yankelevitz DF, Yip R, Reeves AP, Farooqi A, Xu D, et al. Lung cancers diagnosed at annual CT screening: Volume doubling times. Radiology. 2012;263:578-83.

[25] Choi SH, Lee CH, Kwon SY. Characteristics of lung cancer diagnosed with low dose chest ct screening. Respirology. 2012;17:90.

[26] Xu D, Henschke CI, Yankelevitz DF. Frequency of interimdiagnosed lung cancer in the screening trials. Journal of Thoracic Oncology. 2011;6:S1396-S7.

[27] Steurer J. Periodic CT screening in persons with increased risk reduces mortality in lung cancer. Praxis. 2011;100:1377-8.

[28] Schumacher B. Early diagnosis of lung carcinoma: CT screening for smokers? MMW-Fortschritte der Medizin. 2011;153:17.

[29] Lu F, Belitskaya-Levy I, Owusu-Sarpong Y, Walter D, Rom WN, Goldberg J. Lung nodules and patient characteristics in a high risk lung cancer screening cohort. American Journal of Respiratory and Critical Care Medicine. 2011;183.

[30] Kanda S, Fukushima T, Tateishi K, Agatsuma T, Koizumi T, Kubo K, et al. Comparative analysis in patients with small-cell lung cancer (SCLC) detected by chest computed tomography (CT) and X-ray mass-screenings. Journal of Thoracic Oncology. 2011;6:S1346.

[31] Henschke CI, Boffetta P, Gorlova O, Yip R, DeLancey JO, Foy M. Assessment of lung-cancer mortality reduction from CT Screening. Lung Cancer. 2011;71:328-32.

[32] Dhopeshwarkar MR, Roberts HC, Paul NS, Dong Z, Tsao M, Menezes RJ. Screen-detected lung cancer. A retrospective analysis of CT appearance. Academic Radiology. 2011;18:1270-6.

[33] Weiss J. Lung cancer risk/Screening - CT findings in patients with increased cancer risk. Pneumologie. 2010;64:140.

[34] Marshall H, Bowman RV, Crossin J, Fuentes M, Slaughter R, Passmore L, et al. Queensland lung cancer screening study: Findings from the first 197 participants' prevalence low dose CT scans. Respirology. 2010;15:A14.

[35] Croswell JM, Baker SG, Marcus PM, Clapp JD, Kramer BS. Cumulative incidence of false-positive test results in lung cancer screening: A randomized trial. Annals of Internal Medicine. 2010;152:505-12.

[36] Choi SH, Lee CH, Kwon SY. Lung cancer characteristics with low dose chest CT screening in Korea: Comparison with korean general population. Respirology. 2010;15:66.

[37] Akporobaro A, Patel D, Chinyanganya N, Butawan R, Hackshaw A, Seale C, et al. Screening for lung cancer: A qualitative study of the acceptability of screening and participation in the lung-search trial. Thorax. 2010;65:A166-A7.

[38] Ahn MI, Gleeson TG, Chan IH, McWilliams AM, MacDonald SL, Lam S, et al. Perifissural nodules seen at CT screening for lung cancer. Radiology. 2010;254:949-56.

[39] NCI-sponsored study finds increased risk of false-positives in CT lung cancer screening. ONCOLOGY. 2010;24.

[40] Murray CP, Wong PM, Louw J, Waterer GW. Western Australian cigarette smokers have fewer small lung nodules than North Americans on CT screening for lung cancer. Journal of Medical Imaging and Radiation Oncology. 2009;53:339-44.

[41] Grodzki T, Walecka A, Fabian W, Daniel B, Witkiewicz I, Jarmolinski T, et al. Program of early detection of pulmonary neoplasms by the computed tomography - preliminary Szczecin experience. Pneumonologia i alergologia polska : organ Polskiego Towarzystwa Ftyzjopneumonologicznego, Polskiego Towarzystwa Alergologicznego, i Instytutu Gru?licy i Chor?b P?uc. 2009;77:521-7.

[42] Edey AJ, Hansell DM. CT lung cancer screening in the UK. British Journal of Radiology. 2009;82:529-31.

[43] Ronge R. CT screening improves early diagnosis of lung cancer. Pneumologie. 2008;62:185.

[44] Ronge R. CT screening for lung cancer at 1 year intervals in patients with small lung nodules. Tumor Diagnostik und Therapie. 2008;29:65.

[45] Kakinuma R, Moriyama N, Kaneko M, Ohmatsu H, Eguchi K. Practice of low-dose helical CT screening for lung cancer. Respiration and Circulation. 2008;56:457-63.

[46] Welch HG, Woloshin S, Schwartz LM, Gordis L, Gotzsche PC, Harris R, et al. Overstating the evidence for lung cancer screening: The International Early Lung Cancer Action Program (I-ELCAP) study. Archives of Internal Medicine. 2007;167:2289-95.

[47] Markowitz SB, Miller A, Miller J, Manowitz A, Kieding S, Sider L, et al. Ability of low-dose helical CT to distinguish between benign and malignant noncalcified lung nodules. Chest. 2007;131:1028-34.

[48] Henschke CI, Yankelevitz DF, McCauley DI, Rifkin M, Fiore ES, Austin JHM, et al. CT screening for lung cancer: Diagnoses resulting from the New York Early Lung Cancer Action Project. Radiology. 2007;243:239-49.

[49] Carter D, Vazquez M, Flieder DB, Brambilla E, Gazdar A, Noguchi M, et al. Comparison of pathologic findings of baseline and annual repeat cancers diagnosed on CT screening. Lung Cancer. 2007;56:193-9.

[50] Libby DM, Wu N, Lee IJ, Farooqi A, Smith JP, Pasmantier MW, et al. CT screening for lung cancer: The value of short-term CT follow-up. Chest. 2006;129:1039-42.

[51] Henschke CI, Shaham D, Yankelevitz DF, Kramer A, Kostis WJ, Reeves AP, et al. CT screening for lung cancer: Significance of diagnoses in its baseline cycle. Clinical Imaging. 2006;30:11-5.

[52] Townsend CO, Clark MM, Jett JR, Patten CA, Schroeder DR, Nirelli LM, et al. Relation between smoking cessation and receiving results from three annual spiral chest computed tomography scans for lung carcinoma screening. Cancer. 2005;103:2154-62.

[53] Chek K, Tribuna J, Nashelsky J. Is yearly chest x-ray screening helpful in reducing mortality for smokers? Journal of Family Practice. 2005;54:815-6.

[54] McWilliams A, Mayo J, MacDonald S, Leriche JC, Palcic B, Szabo E, et al. Lung Cancer Screening: A Different Paradigm. American Journal of Respiratory and Critical Care Medicine. 2003;168:1167-73.

[55] Li F, Sone S, Abe H, MacMahon H, Doi K. Low-dose computed tomography screening for lung cancer in a general population: Characteristics of cancer in non-smokers versus smokers. Academic Radiology. 2003;10:1013-20.

[56] Schnoll RA, Miller SM, Unger M, McAleer C, Halbherr T, Bradley P. Characteristics of female smokers attending a lung cancer screening program: A pilot study with implications for program development. Lung Cancer. 2002;37:257-65.

[57] Murakami T, Matsuda K, Yasuhara Y, Ikezoe J, Mogami H, Eguchi K, et al. New mass screening project for lung cancer with mobile spiral computed tomography (CT) & computed radiography (CR) in Ehime District. Japanese Journal of Clinical Radiology. 2002;47:128-34.

[58] Henschke CI, Yankelevitz DF, Libby DM, McCauley D, Pasmantier M, Altorki NK, et al. Early Lung Cancer Action Project: Annual screening using single-slice helical CT. 2001. p. 124-34.

[59] Henschke CI, Naidich DP, Yankelevitz DF, McGuinness G, McCauley DI, Smith JP, et al. Early Lung Cancer Action Project: Initial findings on repeat screening. Cancer. 2001;92:153-9.

[60] Henschke CI, McCauley DI, Yankelevitz DF, Naidich DP, McGuinness G, Miettinen OS, et al. Early lung cancer action project: A summary of the findings on baseline screening. Oncologist. 2001;6:147-52.

[61] Henschke CI. Early lung cancer action project: Overall design and findings from baseline screening. Cancer. 2000;89:2474-82.

[62] Kusunoki Y. Diagnosis of small peripheral lung cancers using spiral computed tomography lung cancer screening. Nippon Geka Gakkai zasshi. 1999;100:705-11.

[63] Gartenschlager M, Schweden F, Gast K, Westermeier T, Kauczor H, von Zitzewitz H, et al. Pulmonary nodules: detection with low-dose vs conventional-dose spiral CT. European radiology. 1998;8:609-14.

[64] Itoh S, Satake H, Katoh T, Isomura T, Ikeda M, Ishigaki T. Helical CT for lung-cancer screening: fourth report. Detectability of pulmonary lesions. Nihon Igaku H?shasen Gakkai zasshi Nippon acta radiologica. 1996;56:1039-44.

[65] Naidich DP, Marshall CH, Gribbin C, Arams RS, McCauley DI. Low-dose CT of the lungs: Preliminary observations. Radiology. 1990;175:729-31.

Less than 10 detected lung cancers (n=25)

[1] Bastarrika G, Pueyo JC, Lozano MD, Montuenga L, Zulueta JJ. Screening for lung cancer with low-dose spiral CT: Results in 150 asymptomatic subjects. Medicina Clinica. 2003;121:41-7.

[2] Bensadoun E, Brooks M, Baron A, Mannino DM, Hirschowitz EA, Weaver T, et al. Marty driesler lung cancer project: Preliminary report of lung cancer screening in rural kentucky. American Journal of Respiratory and Critical Care Medicine. 2011;183.

[3] Cardinale L, Cortese G, Borasio P, Dogliotti L, Ferraris F, Novello S, et al. Low dose CT in early lung cancer diagnosis: Prevalence data. Radiologia Medica. 2005;110:532-43.

[4] Dominioni L, Imperatori A, Rovera F, Ochetti A, Paolucci M, Dionigi G. Lung cancer screening in cigarette smokers in the province of Varese, Italy. Cancer. 2000;89:2345-8.

[5] Eun Woo L, Jung Hwan L, Dong Ryeol C, Sung Soo K, Yong Gik L. Lung cancer screening with low dose spiral CT and autofluorescence bronchoscopy in high risk group. European Journal of Cancer, Supplement. 2009;7:517.

[6] Field JK, Raji OY, Cassidy A, Mimnagh C, Hughes J, Warburton C, et al. A pilot lung cancer early detection study in a primary care practice in knowsley, merseyside: The liverpool lung project primary care implementation programme (llppcip). Journal of Thoracic Oncology. 2011;6:S1393.

[7] Franceschini JP, Pereira JR, Kay FU, Chate RC, Costa Junior AS, Trajano ALC, et al. One year's experience with lung cancer screening in Brazil: BRELT1 preliminary results. Innovations: Technology and Techniques in Cardiothoracic and Vascular Surgery. 2014;9:210.

[8] Gupta NK, Freeman RK, Storey S, Reeves D, Ascioti A, Davis C, et al. Lung cancer screening in high-risk individuals with annual low-dose chest CT in a community setting. Journal of Clinical Oncology. 2014;32.

[9] Jones GC, Saunders G, Choe J. Initial results of a pilot program for low-dose CT lung cancer screening in a community radiation oncology practice. International Journal of Radiation Oncology Biology Physics. 2014;90:S576-S7.

[10] Ju SM, Park HB, Kang H, Park CW, Kim W, Seon HJ, et al. Prevalence of non-calcified pulmonary nodules in screening chest computed tomography. Thoracic Cancer. 2013;4:405-9.

[11] Kasuga I, Kubota K, Maezawa H, Takahashi D, Wakabayashi K, Ohtsubo O, et al. Early detection of mediastinal tumors using low-dose spiral computed tomography. Journal of Thoracic Oncology. 2013;8:S981.

[12] Kiszka K, Rudnicka-Sosin L, Tomaszewska R, Urbanczyk-Zawadzka M, Krupinski M, Pikul P, et al. Morphological characteristics of potentially malignant pulmonary nodules in high-risk male smokers detected in lung cancer screening trial in Cracow, Poland. Polish Journal of Pathology. 2013;64:114-20.

[13] Lam VK, Miller M, Dowling L, Singhal S, Young RP, Cabebe EC. Low-dose CT lung cancer screening in the community: A prospective cohort study. Journal of Clinical Oncology. 2014;32.

[14] Lei Y, Chen BJ, Zeng L, Li WM. Application value of low-dose computed tomography for the screening of lung-cancer in high-risk group. Journal of Sichuan University (Medical Science Edition). 2012;43:584-7.

[15] MacRedmond R, McVey G, Lee M, Costello RW, Kenny D, Foley C, et al. Screening for lung cancer using low dose CT scanning: Results of 2 year follow up Thorax. 2006;61:54-6.

[16] Marshall HM, Bowman R, Crossin J, Fuentes M, Slaughter R, Passmore L, et al. Lung cancer screening: Baseline results from australian low dose computed tomography study. Journal of Thoracic Oncology. 2011;6:S515-S6.

[17] Milch H, Kaminetzky M, Pak P, Godelman A, Shmukler A, Koenigsberg TC, et al. Computed tomography screening for lung cancer preliminary results in a diverse urban population. Journal of Thoracic Imaging. 2015;30:157-63.

[18] Mohamed-Hussein A, Ibrahim ME. Evaluation of lung cancer screening practices of chest physicians in Egypt: A pilot national survey. Chest. 2014;146.

[19] Moizs M, Bajzik G, Lelovics Z, Rakvacs M, Strausz J, Repa I. Preliminary experiences with low-dose computed tomography for lung cancer screening in Hungary. Orvosi Hetilap. 2014;155:383-8.

[20] Nahorecki A, Chabowski M, Kuzniar T, Kedzierski B, Jazwiec P, Szuba A, et al. Low-dose computer tomography as a screening tool for lung cancer in a high risk population. 2015. p. 31-7.

[21] Stephenson SM, Mech KF, Sardi A. Lung cancer screening with low-dose spiral computed tomography. The American surgeon. 2005;71:1015-7.

[22] Triphuridet N, Singharuksa S, Sangfai O, Vidhayarkorn S, Leelapisut T, Nantavithya P, et al. Lung cancer detection by low-dose computerized tomography (LDCT) and digital tomosynthesis (DT) for lung cancer screening in a high-risk population: A comparison of detection methods. Journal of Clinical Oncology. 2014;32.

[23] Triphuridet N, Singharuksa S, Sricharunrat T. Screening of lung cancer by low-dose CT (LDCT), digital tomosynthesis (DT) and chest radiography (CR) in a high risk population: A comparison of detection methods. Journal of Thoracic Oncology. 2013;8:S148-S9.

[24] Yasuda S, Shohtsu A, Ohkubo K, Yamada O, Yoshimi Y, Takagi S, et al. Lung cancer screening with spiral CT. Japanese Journal of Lung Cancer. 1997;37:433-9.

[25] Yoshimura A, Andoh M, Kudoh S, Watari J, Tajima H, Kumasaki T, et al. A pilot study of lung cancer screening with low-dose spiral CT. Japanese Journal of Lung Cancer. 2000;40:99-105.

Only reported clinical stages (n=4)

[1] Yip R, Henschke CI, Yankelevitz DF, Boffetta P, Smith JP. The impact of the regimen of screening on lung cancer cure: A comparison of I-ELCAP and NLST. European Journal of Cancer Prevention. 2015;24:201-8.

[2] Sanchez-Salcedo P, Berto J, de-Torres JP, Campo A, Alcaide AB, Bastarrika G, et al. Lung Cancer Screening: Fourteen Year Experience of the Pamplona Early Detection Program (P-IELCAP). Archivos de Bronconeumologia. 2015;51:169-76.

[3] International Early Lung Cancer Action Program Investigators, Henschke CI, Yankelevitz DF, Libby DM, Pasmantier MW, Smith JP, et al. Survival of patients with stage I lung cancer detected on CT screening. New England Journal of Medicine. 2008;359:877.

[4] Henschke CI, Yankelevitz DF, McCauley DI, Rifkin M, Fiore ES, Austin JHM, et al. CT screening for lung cancer: Diagnoses resulting from the New York Early Lung Cancer Action Project. Radiology. 2007;243:239-49.
